# A PDE1 inhibitor reduces adipogenesis in mice via regulation of lipolysis and adipogenic cell signaling

**DOI:** 10.1038/s12276-018-0198-7

**Published:** 2019-01-11

**Authors:** Nam-Jun Kim, Jung-Hwan Baek, JinAh Lee, HyeNa Kim, Jun-Kyu Song, Kyung-Hee Chun

**Affiliations:** 1 Department of Biochemistry and Molecular Biology, Yonsei University College of Medicine, 50 Yonsei-ro, Seodaemun-gu, Seoul, Republic of Korea; 2 Brain Korea 21 PLUS Project for Medical Science, Yonsei University, 50 Yonsei-ro, Seodaemun-gu, Seoul, Republic of Korea; 3 Tumor Microenvironment Research Branch, Division of Cancer Biology, National Cancer Center, Goyang, Republic of Korea

**Keywords:** Obesity, Biochemistry

## Abstract

Vinpocetine, a phosphodiesterase (PDE) type-1 inhibitor, increases cAMP and cGMP levels and is currently used for the management of cerebrovascular disorders, such as stroke, cerebral hemorrhage, and cognitive dysfunctions. In this study, we first determined that vinpocetine effectively suppressed adipogenesis and lipid accumulation. However, we questioned which molecular mechanism is involved because the role of PDE in adipogenesis is still controversial. Vinpocetine decreased adipogenic cell signaling, including the phosphorylation of ERK, AKT, JAK2, and STAT3, and adipokine secretion, including IL-6, IL-10, and IFN-α. Interestingly, vinpocetine increased the phosphorylation of HSL, suggesting the induction of the lipolysis pathway. Moreover, vinpocetine increased UCP1 expression via increasing cAMP and PKA phosphorylation. The administration of vinpocetine with a normal-chow diet (NFD) or a high-fat diet (HFD) in mice attenuated body weight gain in mice fed both the NFD and HFD. These effects were larger in the HFD-fed mice, without a difference in food intake. Vinpocetine drastically decreased fat weight and adipocyte cell sizes in gonadal and inguinal white adipose tissues and in the liver in both diet groups. Serum triacylglycerol levels and fasting blood glucose levels were reduced by vinpocetine treatment. This study suggested that vinpocetine prevents adipocyte differentiation through the inhibition of adipogenesis-associated cell signaling in the early stages of adipogenesis. Moreover, upregulating cAMP levels leads to an increase in lipolysis and UCP1 expression and then inhibits lipid accumulation. Therefore, we suggest that vinpocetine could be an effective agent for treating obesity, as well as improving cognition and cardiovascular function in older individuals.

## Obesity: Natural compound limits fat cell production

A compound extracted from the periwinkle plant can limit the over-production of fat cells and may be a useful agent for treating obesity. Being overweight is the result of changes in the size and number of fat cells, or adipocytes, in the body. Scientists are searching for molecules that can limit the growth and replication of adipocytes, but many anti-obesity agents found to date have unpleasant side-effects. Kyung-Hee Chun at Yonsei University in Seoul, South Korea and co-workers examined the effects of 502 naturally occuring compounds on adipocyte differentiation in cell culture. One compound called vincamine, which is safely used to treat vascular diseases in the brain, decreased cell signaling pathways involved in adipocyte generation in mice and also lowered fasting blood glucose levels.

## Introduction

The immoderate accumulation of fat tissue, the result of an imbalance between energy intake and expenditure, causes obesity^1^. Obesity is a major worldwide issue that increases the incidence of type 2 diabetes mellitus, cardiovascular diseases, hepatosteatosis, hyperlipidemia, and other chronic diseases^2,3^ Obesity is the result of a change in both adipocyte number and the size of individual fat cells. While adult-onset obesity is generally due to adipocyte hypertrophy (increased cell size), adipocyte hyperplasia (increased adipocyte number) mainly occurs in children and unhealthy obese individuals^4^. Therefore, developing an effective way to ameliorate adiposity is an important goal in metabolic disease research. There have been efforts by many researchers to find anti-obesity therapeutic targets. However, most of the anti-obesity agents reported to date are chemical compounds with side effects, such as diarrhea, vomiting, nausea, and insomnia^5^.

To overcome these side effects, many scientists have sought to identify natural compounds that have anti-adipogenic effects^6,7^. In this study, we used a screening approach to identify natural compounds with anti-obesity effects. First, we purchased a library of 502 natural compounds and administered them to cultured pre-adipocytes at 25 μg/ml to study their effects on adipocyte differentiation, especially on the second day of treatment. We selected anti-obesity compounds through Oil Red O (ORO) staining. Among the screened compounds, we excluded compounds known to act as tumor inducers, anti-cancer drugs, and blood pressure reducers as well as compounds with cytotoxicity. Finally, we identified that vinpocetine has anti-obesity effects based on the results of ORO staining.

Vinpocetine is a semisynthetic derivative of vincamine, an alkaloid first extracted from the periwinkle plant during the late 1960s^8,9^. It has been demonstrated that vinpocetine is a phosphodiesterase type-1 (PDE1) inhibitor that increases intracellular cGMP and cAMP concentrations, thereby activating protein kinase A and protein kinase G (PKG), respectively^10–12^. Vinpocetine relaxes cerebral smooth muscle cells and ameliorates cerebral blood flow^13,14^. Considering its favorable effects of vasodilation and neuro-protection, vinpocetine has been widely used for decades in the treatment of cerebrovascular diseases. Vinpocetine ameliorates neuroplasticity via an increase in Ca^2+^ and cAMP/cGMP levels, inducing a cascade that causes the phosphorylation and activation of cAMP responsive element binding protein (CREB) and serum response factor (SRF). Activation of CREB or SRFs promotes the expression of neuroplasticity-associated genes^15^. Recently, vinpocetine was reported to inhibit the proliferation of vascular smooth muscle cells and breast cancer cells^16^. Furthermore, vinpocetine was identified as a potent anti-inflammatory agent. It prohibits NFκB transcription after stimulation of TNFα by blocking the IκB kinase complex (IKK)^17^. However, the anti-obesity effects of vinpocetine have not previously been investigated.

In this study, we demonstrated the molecular mechanisms by which vinpocetine affects adipocyte differentiation and lipid accumulation. We also administered vinpocetine to mice on a high-fat diet (HFD) and determined its anti-obesity effect in vivo. Taken together, we suggest that vinpocetine could be a potent agent for obesity treatment.

## Materials and methods

### Chemicals

An FDA-approved natural product chemical library was purchased from Enzo Life Sciences (Farmingdale, NY). Vinpocetine, isobutylmethylxanthine, dexamethasone, insulin, and ORO powder were purchased from Sigma Chemicals (St. Louis, MO).

### Cell culture

3T3-L1 cells were maintained and differentiated as described previously^18^. Briefly, serum and media were purchased from Thermo Fisher Scientific (Waltham, MA). 3T3-L1 cells were cultured in Dulbecco’s modified Eagle’s medium (DMEM) containing 10% calf serum until confluence. Then, differentiation was induced in DMEM containing 10% FBS, insulin (1 μg/ml), isobutylmethylxanthine (520 μM), and dexamethasone (1 μM) (MDI). After 2 days, the medium was replaced with DMEM supplemented with 10% FBS and insulin (1 μg/ml). Then, DMEM containing 10% FBS without insulin was replaced every 2 days until day 6.

### Cell viability assay

3T3-L1 cells were plated in 12-well plates and incubated until confluence. Then, confluent 3T3-L1 cells were treated with vinpocetine for the indicated time. Cell viability was measured using EZ-Cytox (Daeil Lab Services, Wonju, Korea) according to the manufacturer’s protocol^19,20^.

### Western blot assay

Cell lysate extractions were prepared with radioimmunoprecipitation assay buffer (1% Triton X-100, 1% sodium deoxycholate, 0.1% sodium dodecyl sulfate, 150 mM NaCl, 50 mM Tris–HCl, pH 7.5, and 2 mM EDTA, pH 8.0) as described previously^21–23^. Antibodies against C/EBPα, C/EBPβ, PPARγ, A-FABP, FASN, AKT, p-AKT, AMPKα1/2, p-AMPKα1/2, JAK2, p-JAK2, HSL, and PGC1α were purchased from Santa Cruz Biotechnology (Dallas, TX). Antibodies against ERK, p-ERK, STAT3, p-STAT3, p-HSL, PKA, p-PKA, and UCP1 were obtained from Cell Signaling Technology (Danvers, MA). The normalization control was anti-β-actin (Santa Cruz Biotechnology).

### cAMP direct immunoassay

Differentiated 3T3L1 cells were incubated in 0.1 M HCl for 10 min at room temperature to detach cells from the plate. The cAMP level was measured from supernatants using an ELISA kit in accordance with the manufacturer’s instructions (Enzo Life Science, Farmingdale, NY).

### Oxygen consumption rate (OCR) assay

3T3-L1 cells were seeded in an XF24 microplate and treated with vinpocetine at 2 days after MDI treatment. After 3 days, the XF24 microplate was incubated in a non-CO_2_ incubator for 1 h, and respiration was measured under basal conditions, followed by the addition of 2.6 μM oligomycin, 0.5 μM FCCP, and 1 μM rotenone/antimycin A. OCRs were measured using an XF24 analyzer (Seashore Bioscience).

### ORO staining

Differentiated 3T3-L1 cells were incubated with 10% formalin for 10 min and washed with distilled water. Then, the cells were stained with ORO in 60% isopropanol. Finally, the ORO stain that bound to the cells was eluted with 100% isopropanol and measured using the OD_500_.

### RNA isolation, reverse transcription-polymerase chain reaction (RT-PCR), and quantitative RT-PCR (qPCR) analysis

For the RT-PCR and qPCR experiments, total mRNA was isolated from tissue samples and cultured cells using an RNA lysis reagent (easy-BLUE™, iNtRON Biotechnology, Seongnam, Korea) according to the manufacturer’s instructions. cDNA (1 μg) was synthesized from RNA using a qPCR RT master mix (Takara Bio, Otsu, Japan). Real-time RT-PCR was performed using SYBR^®^ Green master mix and a thermal cycler, both of which were purchased from Applied Biosystems (Foster City, CA)^21–23^. The primers used are described in Supplementary Figure S1.

### Mice and diets

Wild-type C57BL6/J male mice were purchased from Orient Bio Inc. (Gyeonggi, Korea) (permission number for animal experiments: 2015-0094). For treatment, 10 and 20 mg/kg vinpocetine were intraperitoneally injected. For assessing metabolic parameters, 2-month-old mice were fed either a normal-chow diet (NFD) or a high-fat chow diet (HFD, 60% of kcal as fat, OpenSource Diets, New Brunswick, NJ) with or without vinpocetine for 10 weeks as described previously^18^. To confirm the effects of vinpocetine treatment, parameters, such as body weight and food intake were measured. All mouse tissues were frozen in liquid nitrogen and stored at −80 °C before performing experiments.

### Analysis of serum metabolic parameters

Blood was drawn from the mice as mentioned above and centrifuged for 10 min at 132 × *g* to obtain serum. Serum analysis was performed at Seoul Medical Science Institute, Korea.

### Metabolic assays

To perform glucose tolerance tests (GTTs), 1 g/kg glucose (Sigma-Aldrich, St. Louis, MO) was intraperitoneally injected into the mice, whereas 1 U/kg insulin (Humulin) was intraperitoneally administered into the mice for the insulin tolerance tests (ITTs). The blood glucose level was measured at 0, 15, 30, 60, 90, and 120 min after injection.

### Morphological analysis of tissues

The gonadal white adipose tissue (gWAT), inguinal white adipose tissue (iWAT), liver, and brown adipose tissue (BAT) were fixed in 4% paraformaldehyde for 24–48 h at 4 °C, processed for paraffin embedding, and stained with hematoxylin and eosin (H&E). Cell size was analyzed using the ImageJ software program.

### Statistical analysis

We employed unpaired *t*-tests and repeated measures ANOVA to analyze comparisons between two groups. Statistical analysis was performed using Prism 5 (GraphPad, La Jolla, CA). *P*-values < 0.05 were considered to be significant.

## Results

### Vinpocetine inhibits the differentiation of 3T3-L1 cells and the expression of adipogenesis-associated factors

**Fig. 1:**
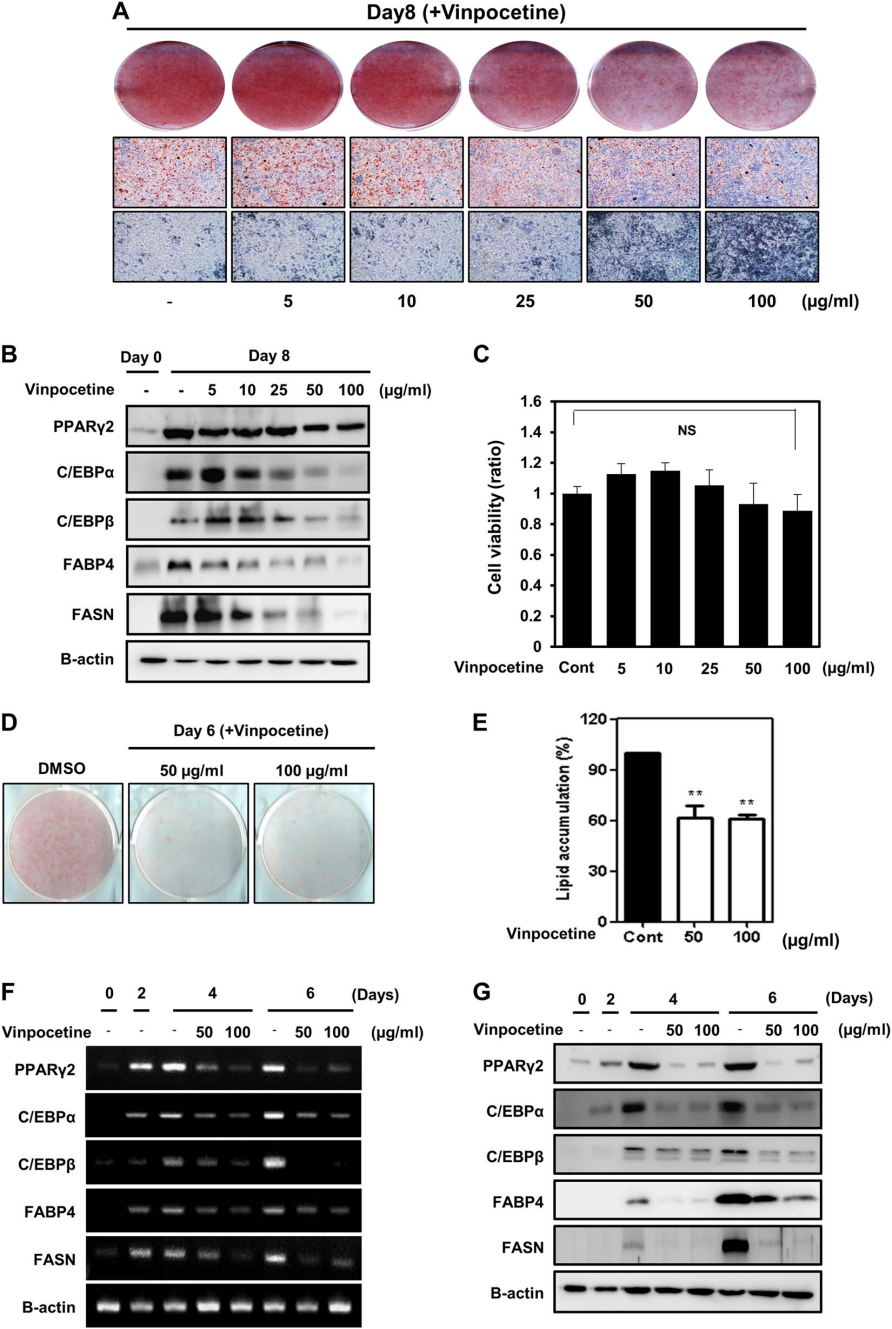
Effects of vinpocetine on MDI-induced adipogenesis in 3T3-L1 cells. **a** 3T3-L1 cells were treated with 0, 5, 10, 25, 50, or 100 μg/ml vinpocetine at 2 days after MDI treatment. Then, Oil Red O staining was performed on day 6 as described in the “Materials and methods”, and images were acquired by microscopy. **b** Vinpocetine was administered to 3T3-L1 cells at a range of doses, and cell lysates were harvested at 0 and 6 days after inducing differentiation. Protein expression of PPARγ, C/EBPα, C/EBPβ, and downstream factors, such as FABP4 and fatty acid synthase in 3T3-L1 cells was detected by western blotting. β-actin was used as a load control. **c** Cytotoxicity in 3T3-L1 preadipocytes treated with up to 100 μg/ml vinpocetine was measured by MTT assay (*n* = 3 for each lane). **d** 3T3-L1 cells were treated with vinpocetine at 50 or 100 μg/ml, and Oil Red O staining was performed as described in the “Materials and methods”. Cells were differentiated for 6 days. **e** Lipid accumulation in 3T3-L1 cells was measured using spectrophotometry as described in the “Materials and methods” (*n* = 3 for each lane). **f**, **g** 3T3-L1 cells were treated with 50 or 100 μg/ml vinpocetine at 2 days after MDI treatment, and cell lysates were harvested at days 0, 2, 4, and 6 after inducing the differentiation of the cells. The mRNA expression levels of the genes encoding adipogenesis-associated factors, such as PPARγ, C/EBPα, and C/EBPβ, and downstream factors, such as FABP4 and fatty acid synthase, were detected by RT-PCR analysis. The protein expression of adipogenesis-associated factors and downstream factors was detected by western blotting. β-actin was used as a load control. Data are presented as the mean ± standard error of the mean (SD); ns = not significant; **P < 0.01 for untreated control vs. 50 and 100 μg/ml vinpocetine

To confirm the effect of vinpocetine on lipid accumulation, cells were treated with different doses of vinpocetine at 2 days after MDI treatment. Adipogenesis was measured by ORO staining of lipid droplets and microscopic imaging 6 days after MDI treatment. ORO staining and microscopy results showed that treatment with vinpocetine reduced 3T3-L1 cell differentiation in a dose-dependent manner. Specifically, 50 and 100 μg/ml vinpocetine were the most effective doses for inhibiting adipocyte differentiation (Fig. 1a). Vinpocetine also decreased the protein expression levels of PPARγ, C/EBPα, and C/EBPβ (three master regulators of adipogenesis) and FASN and FABP4 (downstream factors of PPARγ) (Fig. 1b). To confirm that the inhibition of adipocyte differentiation was not due to attenuated cell viability, a cell viability assay was performed. The effect of vinpocetine on adipocyte differentiation was not attributable to a decline in cell viability (Fig. 1c).

We performed ORO staining of the cells treated with 50 and 100 μg/ml vinpocetine, and the results showed that vinpocetine effectively attenuated MDI-mediated 3T3-L1 cell differentiation (Fig. 1d). Furthermore, vinpocetine-treated adipocytes had 40% less lipid accumulation than did the dimethyl sulfoxide-treated control adipocytes (Fig. 1e). Consistently, the protein and mRNA expression levels of PPARγ, C/EBPα, and C/EBPβ, as well as FABP4 and FASN were markedly reduced in 3T3-L1 cells after vinpocetine treatment (Fig. 1f, g). Collectively, vinpocetine inhibits adipocyte differentiation by decreasing the expression of adipogenesis-associated proteins and genes.

### Vinpocetine retards adipocyte differentiation by inhibiting adipogenesis-associated cell signaling at an early stage and inducing lipolysis and UCP1 expression at a late stage

**Fig. 2:**
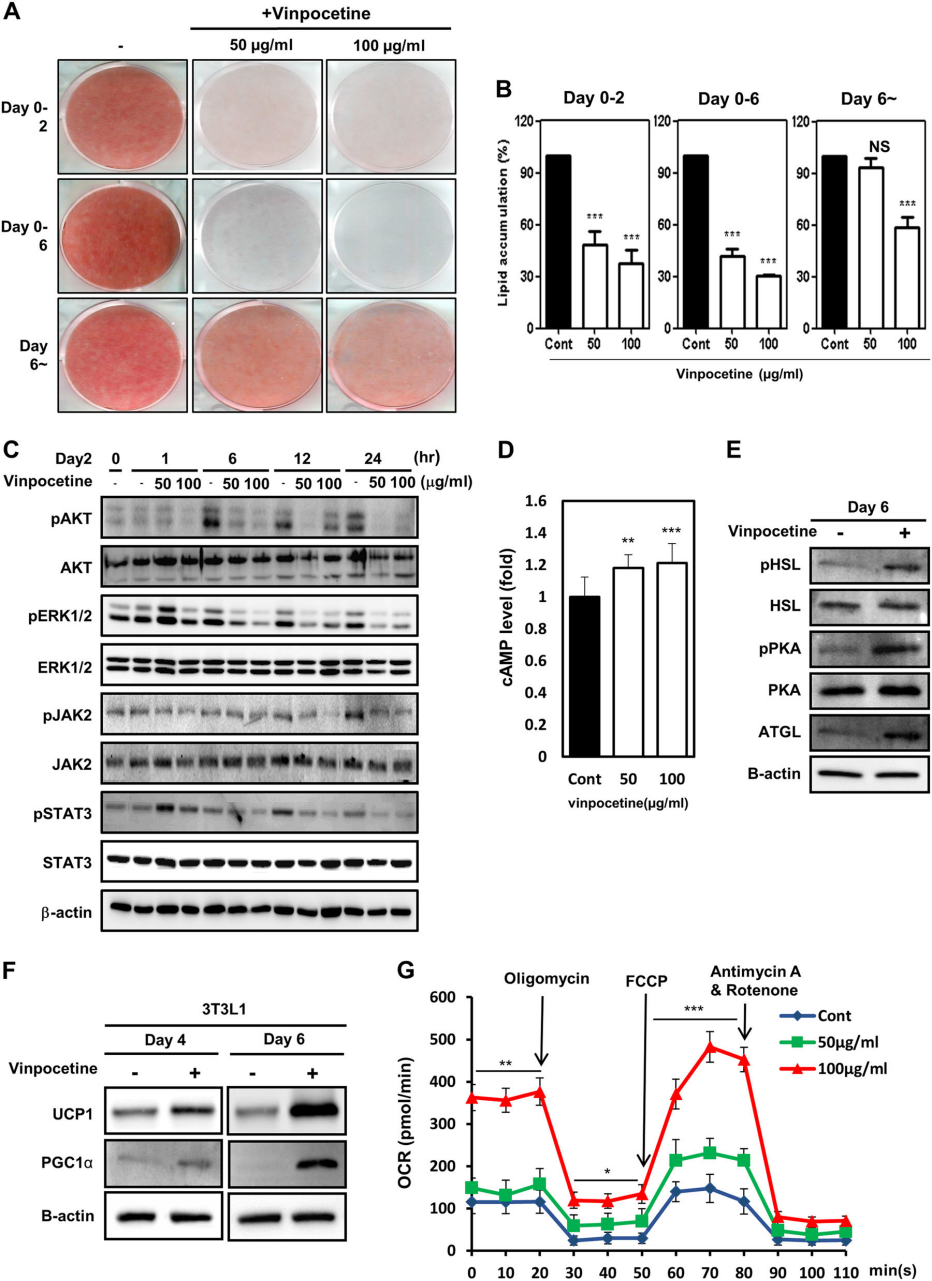
Treatment with vinpocetine retards adipocyte differentiation by inhibiting adipogenesis-associated cell signaling at early stages and inducing lipolysis and UCP1 expression at late stages. **a** 3T3-L1 cells were treated with 50 or 100 μg/ml vinpocetine from day 0, and Oil Red O staining was performed as described in the “Materials and methods” on day 2, day 6, or after day 6 of adipocyte differentiation. **b** 3T3-L1 cells were treated with 50 or 100 μg/ml vinpocetine, and its effects on the early and late stages of 3T3-L1 differentiation and lipid accumulation were measured using spectrophotometry as described in the “Materials and methods” (*n* = 3 for each lane). **c** 3T3-L1 cells were treated with 50 or 100 μg/ml vinpocetine on day 2 after inducing differentiation, and cell lysates were harvested at 0, 1, 6, 12, and 24 h. The phosphorylated forms of AKT, ERK, JAK2, and STAT3 were detected by western blotting. β-actin was used as a load control. **d**, **e** 3T3-L1 cells were treated with vinpocetine, and cell lysates were harvested at 6 days after MDI induction. **d** The cAMP level was measured using a cAMP direct immunoassay kit as described in the “Material and methods” (*n* = 3 for each lane). **e** The phosphorylated and total forms of ATGL, PKA, and HSL were detected by western blotting. **f** 3T3-L1 cells were treated with 100 μg/ml vinpocetine, and cell lysates were harvested at days 4 and 6. The protein expression of UCP1 and PGC-1α was detected by western blotting. β-actin was used as a load control. **g** OCR was measured in differentiated 3T3-L1 cells in the presence of 2.6 μM oligomycin, 0.5 μM FCCP, and 1 μM rotenone/antimycin A (*n* = 3 for each lane). Data are presented as the mean ± SD; *P < 0.05; **P < 0.01; and ***P < 0.001 for untreated control vs. 50 and 100 μg/ml vinpocetine

We attempted to further investigate whether the inhibitory effect of vinpocetine works at the early or late stage of adipogenesis. The ORO staining results showed that vinpocetine retarded all stages of adipocyte differentiation, including the early stage (day 0 to day 2), the late stage (day 0 to day 6), and after day 6 (Fig. 2a). Consistently, microscopy images showed that vinpocetine hindered both the early and late stages of adipocyte differentiation (Supplementary Figure S2A). Additionally, lipid accumulation was reduced by vinpocetine at both the early and late stages of differentiation (Fig. 2b). Furthermore, we investigated how vinpocetine attenuates adipocyte differentiation and lipid accumulation in terms of mode of function. Cellular signaling molecules, such as ERK, AKT, AMPK, and JAK2-STAT3, are known to regulate lipid formation and adipogenesis^32^. Therefore, we aimed to survey whether vinpocetine regulates these signaling pathways. Vinpocetine effectively attenuated the phosphorylation of AKT, ERK, and JAK2-STAT3, but not AMPK, at 4 days after MDI induction (Supplementary Figure S2b). We then investigated whether vinpocetine prohibited the phosphorylation of signaling molecules from day 2 to day 3 of adipocyte differentiation (Fig. 2c). These results showed that vinpocetine reduced the phosphorylation of adipogenesis-associated signaling pathways at the early stages of adipocyte differentiation. We also determined that vinpocetine induces the upregulation of cAMP levels and the phosphorylation of lipolysis-associated factors (Fig. 2d, e). We confirmed that the protein expression of UCP1 and PGC-1α, which induces thermogenesis and reduces lipid accumulation^24,25^, is augmented in vinpocetine-treated adipocytes 4 and 6 days after MDI treatment (Fig. 2f). OCRs were also augmented in vinpocetine-treated adipocytes 5 days after MDI induction (Fig. 2g). Taken together, vinpocetine represses adipocyte differentiation by inhibiting adipogenesis-associated cell signaling at the stages of differentiation and inhibits lipid accumulation by inducing the lipolysis pathway and UCP1 expression.

### Vinpocetine-treated mice have reduced body weight, WAT size, and adipogenesis-associated gene expression

**Fig. 3:**
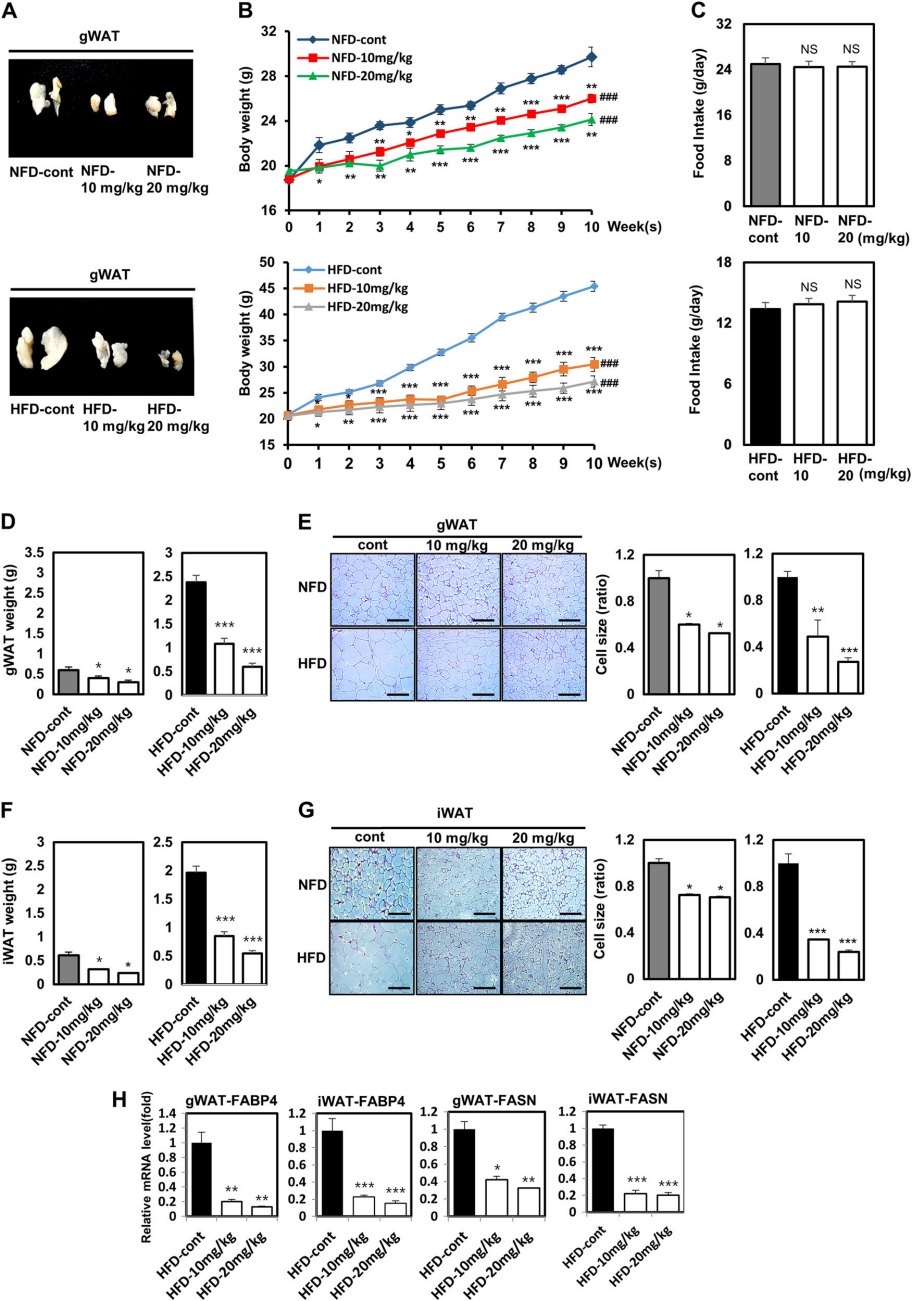
Vinpocetine-treated mice have diminished weight and cell size of gWAT, as well as adipogenesis-associated gene expression. **a** Vinpocetine was administered to both HFD-fed and NFD-fed mice as described in the “Materials and methods”. **b** Body weight was measured using a weighing scale every 2 days. **c** Food intake was measured by weighing the remaining chow. **d** Gonadal WAT weight was measured after the 10-week experimental diet period. **e** The adipocyte size in gonadal WAT sections was determined by staining with hematoxylin and eosin (H&E). Size measurements were performed using ImageJ software. **f** Inguinal WAT weight was measured after the 10-week experimental diet period. **g** The adipocyte size of inguinal WAT sections was determined by staining with H&E. Size measurements were performed as above. **h** Gonadal WAT and inguinal WAT were collected, and tissue lysates were prepared as described in the “Materials and methods”. The mRNA expression of FABP4 and FASN was detected by qPCR analysis. β-actin was used as a normalization control. Data are presented as the mean ± SD; *P < 0.05 and ***P < 0.001 for untreated control vs. 10 and 20 mg/kg vinpocetine (unpaired *t*-tests). ^###^*P* < 0.0001 for untreated control vs. 10 and 20 mg/kg vinpocetine (repeated measures ANOVA)

Next, we investigated the anti-obesity effects of vinpocetine. Mice were fed an NFD or an HFD (containing 60% of kcal as fat) with or without vinpocetine for 10 weeks. As shown by the photographic data, the amount of gWAT was lessened in vinpocetine-treated mice, especially in the HFD-induced obese mice (Fig. 3a). Vinpocetine-treated mice also had lower body weight in both the NFD-fed and HFD-fed groups than that of the untreated control mice (Fig. 3b). The extent of the reduction in body weight was particularly notable in the HFD-fed mice. However, there were no significant differences in food intake between the two diet groups (Fig. 3c).

Moreover, we identified that the vinpocetine-treated mice had a lower gWAT weight in both the NFD-fed and HFD-fed groups (Fig. 3d) and a lower ratio of gWAT weight to body weight than that of the untreated control mice (Supplementary Figure S3). For histological analysis, we performed H&E staining of sectioned gWAT. Reduced adipocyte sizes in gWAT were detected in the vinpocetine-treated NFD-fed and HFD-fed mice (Fig. 3e). We also determined the effect of vinpocetine in iWAT. The vinpocetine-treated mice had lower iWAT weight in both the NFD-fed and HFD-fed groups (Fig. 3f) and a lower ratio of iWAT weight to body weight than that of control mice (Supplementary Figure S4). Moreover, H&E staining data showed that adipocyte size in iWAT in the vinpocetine-treated NFD-fed and HFD-fed mice was reduced (Fig. 3g). Interestingly, the mRNA expression of FABP4 and FASN was reduced in gWAT and iWAT in the vinpocetine-treated HFD-fed mice (Fig. 3h), suggesting that vinpocetine significantly reduces lipid accumulation through the downregulation of FABP4 and FASN in gWAT. Similar to HFD-fed mice, the mRNA expression of FABP4 and FASN was reduced in vinpocetine-treated NFD-fed mice (Supplementary Figure S5). Taken together, vinpocetine reduces the weight of and cell size in both gWAT and iWAT by downregulating adipogenesis-associated gene expression.

### Vinpocetine suppresses lipid accumulation in mouse liver tissue

**Fig. 4:**
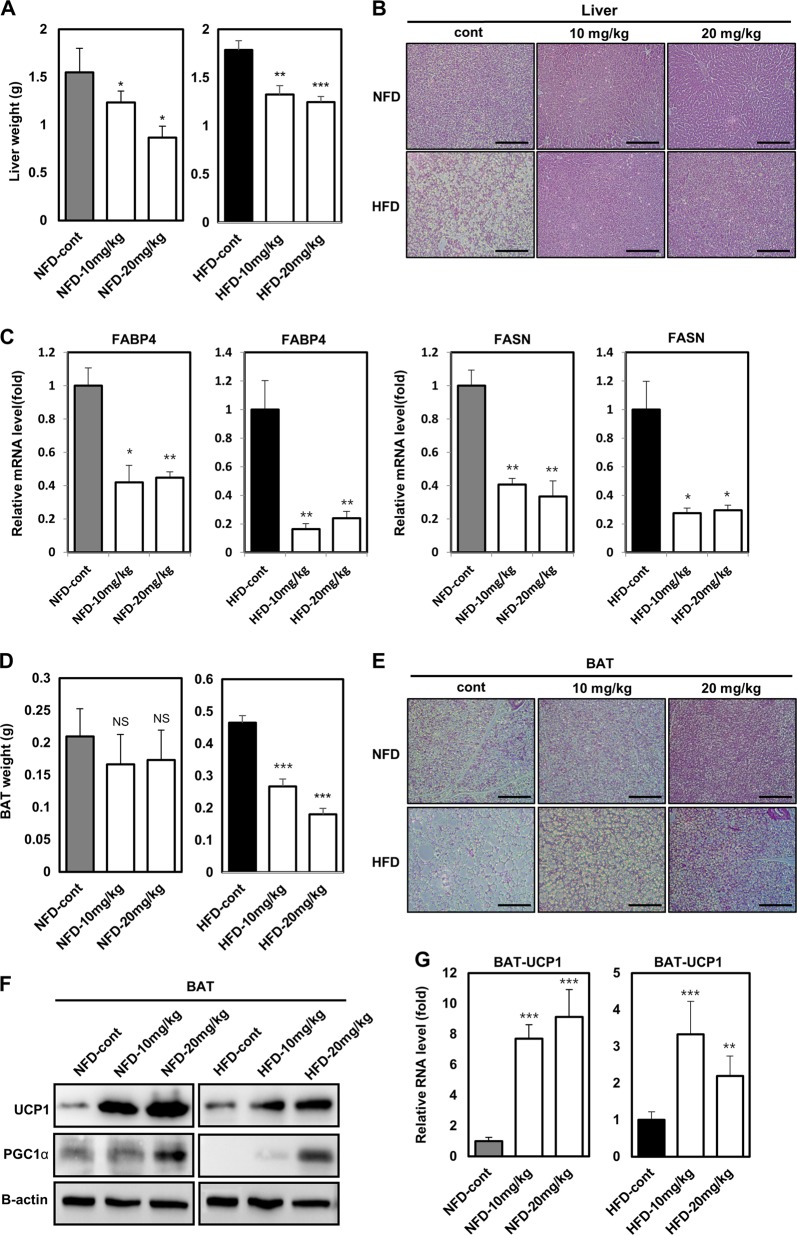
Vinpocetine retards lipid accumulation in mouse liver tissues and BAT. Vinpocetine was intraperitoneally injected into HFD mice, as described in the “Materials and methods”. **a** Both NFD-fed and HFD-fed mice were sacrificed after the 10-week experimental diet period. Then, the liver weight of these mice was measured. **b** H&E staining of liver sections was performed in each indicated group treated with or without vinpocetine for 10 weeks as described in the “Materials and methods”. **c** Liver tissues were collected, and tissue lysates were prepared as described in the “Materials and methods”. The mRNA expression of FABP4 and FASN was detected by qPCR analysis. β-actin was used as a normalization control. **d** Both NFD-fed and HFD-fed mice were sacrificed after the 10-week experimental diet period. Then, the BAT weight was measured using a weighing scale. **e** H&E staining of BAT sections from mice was performed in the indicated group treated with or without vinpocetine for 10 weeks as described in the “Materials and methods.” **f** BAT was collected, and tissue lysate was prepared as described in the “Materials and methods” The protein expression of UCP1 and PGC-1α was detected by western blotting. β-actin was used as a load control. **g** BAT was collected, and tissue lysate was prepared as described in the “Materials and methods”. The mRNA expression of UCP1 was detected by qPCR analysis. β-actin was used as a normalization control. Data are presented as the mean ± SD (*n* = 4 for NFD-fed mice and *n* = 8 for HFD-fed mice); *P < 0.05, **P < 0.01, and ***P < 0.001 for untreated control vs. 10 and 20 mg/kg vinpocetine

We determined whether vinpocetine ameliorates lipid accumulation in the liver tissues of NFD-fed and HFD-fed mice. We identified that the liver weight of vinpocetine-treated mice was lower than that of the control mice, but there were no significant differences in the liver weight to body weight ratio (Fig. 4a, Supplementary Figure S6). Next, we performed H&E staining of sectioned liver tissues. As expected, lipid accumulation in liver tissues was lessened in vinpocetine-treated NFD-fed and HFD-fed mice (Fig. 4b). The expression of adipogenesis-associated genes, including FABP4 and FASN, was attenuated in vinpocetine-treated liver tissues (Fig. 4c). Taken together, vinpocetine suppresses lipid accumulation in liver tissues.

### Vinpocetine induces BAT activation

Similar to its effect in liver tissue, vinpocetine reduced the weight of BAT in HFD-fed mice to a greater degree than that in untreated control HFD-fed mice, whereas it did not affect BAT weight in NFD-fed mice (Fig. 4d). For histological analysis, we performed H&E staining of sectioned BAT tissues. The results indicated that the lipid ratio was reduced in BAT sections from HFD-fed mice (Fig. 4e). However, there were no differences in the ratio of BAT weight to body weight in HFD-fed mice (Supplementary Figure S7).

We confirmed the protein expression of UCP1 and PGC-1α (Fig. 4f) and also analyzed the mRNA expression of UCP1 in BAT (Fig. 4g). Interestingly, vinpocetine significantly increased the protein and mRNA expression of UCP1 in NFD-fed and HFD-fed mice. Taken together, the results indicate that vinpocetine inhibits lipid accumulation by upregulating the expression of UCP1.

### Vinpocetine ameliorates metabolic parameters related to hyperlipidemia, liver function, and glucose homeostasis

**Fig. 5:**
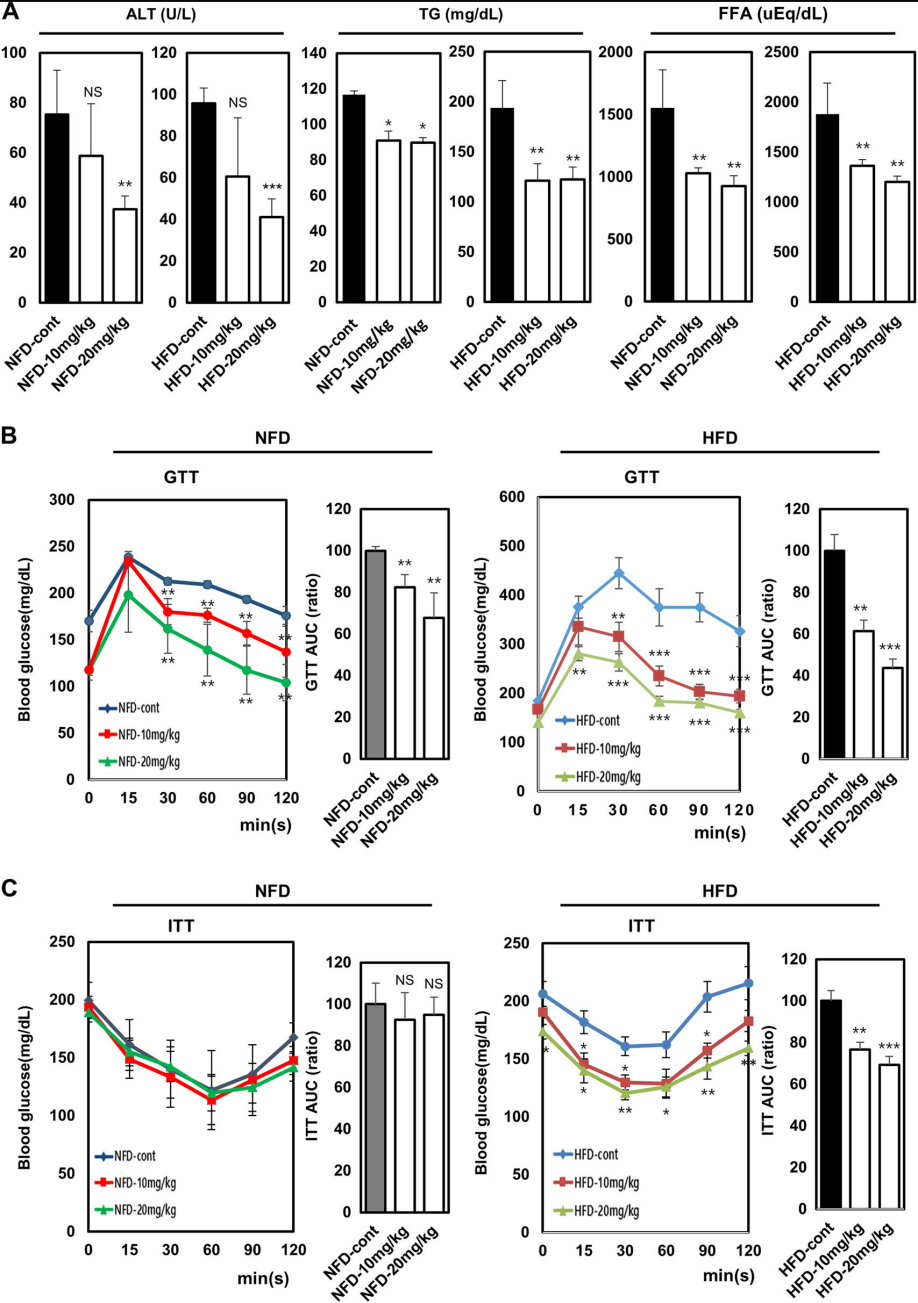
Vinpocetine improves serum metabolic parameters and glucose homeostasis. **a** Serum analyses of TG, FFA, and ALT were performed as described in the “Materials and methods”. **b** For glucose tolerance tests, 1 g/kg glucose was intraperitoneally injected into both the NFD-fed and HFD-fed mice. **c** For insulin tolerance tests, 1 U/kg insulin was intraperitoneally administered into both the NFD-fed and HFD-fed mice. The blood glucose level was measured at different time points as described in the “Material and methods”. Data are presented as the mean ± SD (*n* = 4 for NFD-fed mice and *n* = 8 for HFD-fed mice) *P < 0.05; **P < 0.01; and ***P < 0.001 for untreated control vs. 10 and 20 mg/kg vinpocetine

We determined whether vinpocetine might ameliorate metabolic parameters, such as the levels of TG, FFA as a blood fat, and ALT as a biomarker for liver health in the serum of the experimental mice. As expected, vinpocetine diminished the levels of TG, FAA, and ALT in both the NFD-fed and HFD-fed mice (Fig. 5a). Considering the effects of vinpocetine on glucose homeostasis, we performed GTT assays for 120 min. Clearance of blood glucose was significantly faster in vinpocetine-treated mice than in control mice (Fig. 5b). Insulin sensitivity was analyzed by ITT. There were no differences in blood glucose levels between the vinpocetine-treated NFD-fed mice and the untreated control NFD-fed mice, but the blood glucose level was lower in the vinpocetine-treated HFD-fed mice than in the control HFD-fed mice (Fig. 5c). These data indicate that vinpocetine improves hyperlipidemia, liver function, and glucose homeostasis in HFD-induced obese mice.

### Vinpocetine hampers the secretion of adipokines in vitro and in vivo

**Fig. 6:**
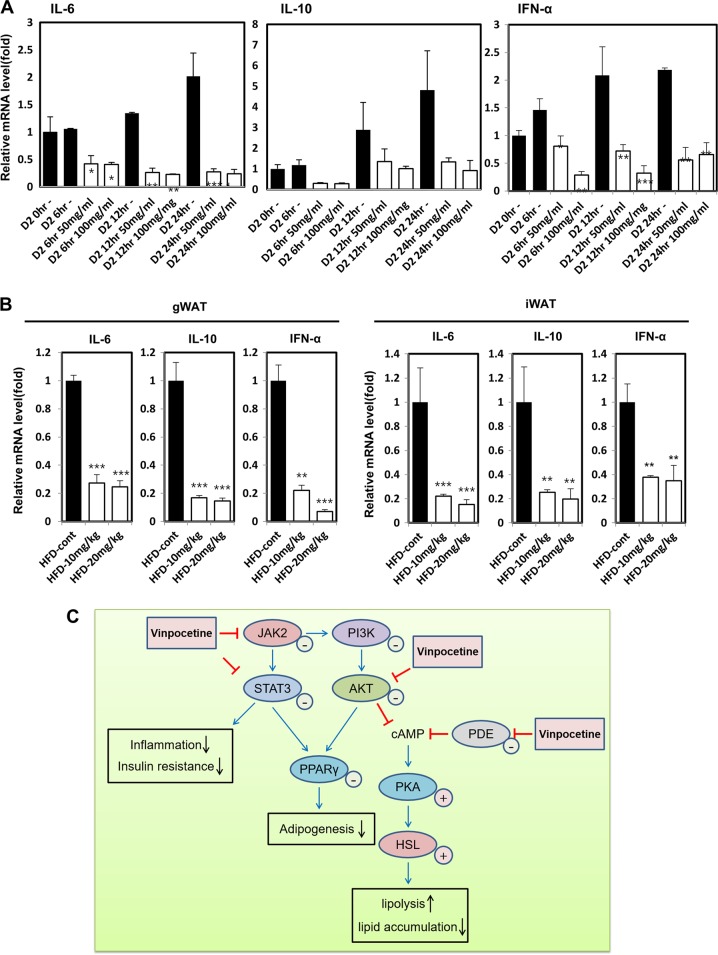
Vinpocetine inhibits the secretion of adipokines in vitro and in vivo. **a** 3T3-L1 cells were treated with 50 or 100 μg/ml vinpocetine on day 2 after inducing differentiation, and cell lysates were harvested at the 6, 12, and 24-h time points. The mRNA expression of Il-10, IL-6, and IFN-α was detected by qPCR analysis. β-actin was used as a normalization control. **b** Vinpocetine was administered to both the HFD-fed and NFD-fed mice, as described in the “Materials and methods”. Gonadal WAT and inguinal WAT were collected, and tissue lysates were prepared as described in the “Materials and methods”. The mRNA expression of IL-10, IL-6, and IFN-α was detected by qPCR analysis. β-actin was used as a normalization control. Data are presented as the mean ± SD; *P < 0.05 and ***P < 0.001 for untreated control vs. 50 and 100 μg/ml vinpocetine. Data are presented as the mean ± SD (*n* = 4 for NFD-fed mice and *n* = 8 for HFD-fed mice); *P < 0.05; **P < 0.01; and ***P < 0.001 for untreated control vs. 10 and 20 mg/kg vinpocetine. **c** Schematic representation of the mechanism of action for vinpocetine

Because we initially identified that vinpocetine inhibited the activation of the STAT3 pathway (Fig. 2d), we hypothesized that vinpocetine might downregulate cytokines, such as IL-6, IL-10, and IFN-α, in 3T3-L1 cells and in the WAT of HFD-fed mice. The mRNA expression of cytokine-encoding genes, including IL-6, IL-10, and IFN-α, was markedly diminished by vinpocetine in 3T3-L1 cells (Fig. 6a) and in the WAT of HFD-fed mice (Fig. 6b). These data show that vinpocetine inhibits the activation of adipokines both in vitro and in vivo.

## Discussion

In this study, we demonstrated that vinpocetine has pronounced effects of inhibiting adipocyte differentiation and reducing lipid accumulation in 3T3-L1 cells in vitro and in HFD-fed mice in vivo (Fig. 6c). Vinpocetine has been widely used as a drug in Eastern Europe for the treatment of cerebrovascular disorders and age-related memory impairment^14,26^, and it is marketed as a supplement for vasodilation and as a nootropic for the improvement of memory^27,28^. Vinpocetine, a derivative of the vinca alkaloid vincamine, is a known PDE1 inhibitor that acts to increase the intracellular levels of cAMP and cGMP, thereby activating protein kinase A and PKG^11,12,15^.

First, we confirmed that vinpocetine exerts an inhibitory effect on adipogenesis-associated cell signaling at the early stage of adipocyte differentiation. However, the cAMP-dependent mechanism is essential during the early stage of adipocyte differentiation. Usually, researchers treat 3T3-L1 cells with 3-isobutyl-1-methylxanthine (IBMX), a nonselective PDE inhibitor, to activate CREB and induce downstream adipogenic factors, such as CCAAT/enhancer-binding protein alpha (C/EBPα) and peroxisome proliferator-activated receptor **γ**^29,30^. This suggests that inhibition of PDE is important to initiate adipogenesis and lipid accumulation in adipocytes. Therefore, we determined which signaling pathways were affected by vinpocetine treatment. Previously, vinpocetine was shown to attenuate the phosphorylation of AKT and ERK1/2, which are molecules related to the MAPK signaling pathway^14,19,31^. As a result, vinpocetine reduced the phosphorylation of AKT and ERK1/2 in the progression of early adipocyte differentiation, but it did not affect the phosphorylation of AMPK. Moreover, it has been reported that rapid activation of JAK2-STAT3 signaling is elicited upon the induction of adipogenesis^32^. STAT3 is activated within 2 h after adipogenic induction, at which point phosphorylated STAT3 translocates from the cytoplasm to the nucleus^33^. Upon the activation of JAK2-STAT3 signaling, STAT3 regulates the transcription of CEBP by binding to the distal region of its promoter in the early stage of adipogenesis. We thought that vinpocetine may act as a STAT3 inhibitor and found that vinpocetine blocks the phosphorylation of JAK2 and STAT3. It is also known that the expression of inflammatory cytokines, such as IL-6, IL-10, and IFN-α is greatly influenced by STAT3 signaling^34,35^. There is an enhanced secretion of some interleukins and inflammatory cytokines in the adipose tissue of obese individuals, as well as an increased circulating level of many cytokines^36^. Adipocytes secrete cytokines such as IL-6, IL-10, and IFN-α^36^. IL-6, IL-10, and IFN-α are among the upstream factors of STAT3. We also identified that the expression of the genes encoding IL-6, IL-10, and IFN-α was inhibited by vinpocetine, suggesting that these cytokines may regulate one another in an autocrine manner. FABP4 is a major target gene of PPARγ and encodes a lipid transporter called aP2^37^. PPARγ induces FABP4 expression in adipocytes and macrophages. FABP4 is implicated in the development of insulin resistance, atherosclerosis, non-alcoholic fatty liver disease, and obesity^37^. In the progression of de novo lipogenesis, FASN catalyzes the de novo synthesis of long-chain fatty acids from acetyl-coenzyme A and malonyl-coenzyme A, and an increase in its expression has been reported in obesity^38,39^. As FABP4 and FASN are involved in lipid accumulation in adipocytes, WAT, and the liver, we investigated whether vinpocetine regulates the expression of FABP4 and FASN. As shown by our data, vinpocetine is likely to reduce the expression of FABP4 and FASN. Previously, we reported that galectin-3 regulates PPARγ and is involved in the formation of WAT^18^. Similarly, we tried to determine whether vinpocetine can reduce WAT formation as well as lipid accumulation in liver tissue. We intraperitoneally administered vinpocetine into both NFD-fed and HFD-fed mice to confirm the attenuation of WAT formation and the reduced expression of FABP4, FASN, and cytokine-encoding genes, such as IL-6, IL-10, and IFN-α. Lipid accumulation in the liver was decreased. These results suggested that vinpocetine blocks adipogenesis initiation through inhibition of adipogenic cell signaling rather than inhibition of PDE.

However, we still have an interest in the role of vinpocetine as a PDE1 inhibitor in adipocytes and lipid accumulation. The PDE family inhibitors block hydrolysis of intracellular cAMP/cGMP, induce transcriptional cascades associated with CREB, and increase or decrease of Ca^2+^ levels^15^. PDE3 inhibitors hydrolyze cGMP and relax vascular and airway smooth muscle, prohibit platelet aggregation and promote lipolysis^40,41^. Additionally, a PDE5 inhibitor has been known to cure erectile dysfunction. The PDE5 inhibitor sildenafil increases the level of intracellular cGMP and triggers penile erection. cGMP activates cGMP-dependent PKG, which phosphorylates several proteins. These phosphorylated proteins cause reduced intracellular Ca^2+^ levels and a relaxation of arterial and trabecular smooth muscle, leading to arterial dilatation, venous constriction, and the rigidity of penile erection^42,43^. We also found that upregulated cAMP levels could increase the phosphorylation of protein kinase (PKA)^44^ and that activated PKA could activate hormone-sensitive lipase (HSL)^45^ and adipose triglyceride lipase (ATGL)^46^. Therefore, we questioned whether PDE1 inhibitors stimulate the lipolysis signal pathway. We determined here that vinpocetine upregulated cAMP levels in adipocyte cells and consequently increased PKA phosphorylation and HSL and ATGL protein levels.

Unlike the lipolysis pathway, we also found that the protein and mRNA levels of UCP1 were increased in vinpocetine-treated adipocytes and BAT. Vinpocetine treatment in adipocytes also increased oxygen consumption, suggesting that vinpocetine considerably triggers BAT activation. It was documented that PDE3 and PED4 isozyme-selective inhibitors increase intracellular cAMP levels, thereby inducing the upregulation of the lipolysis pathway and UCP1 expression^47^. They mentioned that the induction of UCP1 or lipolysis was not altered by the inhibition of PDE1, PDE2, or PDE8A. However, we identified that vinpocetine promotes the lipolysis pathway and UCP1 expression at the late stages of adipocyte differentiation. We hypothesize that the effect of vinpocetine on the increase in UCP1 expression may be caused by the downregulation of adipogenic cell signaling and the upregulation of cAMP levels.

Furthermore, insulin sensitivity was improved by vinpocetine. There is some evidence for the relationship between vinpocetine and glucose homeostasis. PDE1C downregulates glucose-induced insulin secretion^48^. PDE3B-overexpressing mice have insulin resistance, islet dysfunction, and glucose intolerance^49^. Vinpocetine might regulate insulin sensitivity through a PDE inhibitor, as in previous studies. An increase in pro-inflammatory cytokines in adipose tissues is known to induce insulin resistance. Because vinpocetine has an anti-inflammatory effect, insulin resistance could be improved by reducing the level of inflammatory cytokines in adipose tissues. The food intake of NFD-fed and HFD-fed mice did not differ between the vinpocetine-treated and untreated groups. As a result, the effect of vinpocetine was independent of food intake, and there was no suggestion that longer-term vinpocetine treatment would be lethal in mice. Moreover, vinpocetine also reduced the body weight of NFD-fed mice, suggesting that vinpocetine could be a promising agent for the prevention of obesity.

In summary, we demonstrated that vinpocetine attenuates adipogenesis-associated cell signaling pathways at the early stage of differentiation and facilitates lipolysis pathways at the late stage of differentiation. Second, we identified that the UCP1 level is augmented in adipocytes and BAT by vinpocetine. Furthermore, it is likely to reduce body weight by inhibiting FABP4 and FASN expression and cytokine secretion. However, further study is needed to investigate the detailed mechanism underlying the anti-obesity effects of vinpocetine. Taken together, we showed in this study that vinpocetine has significant inhibitory effects on adipocyte differentiation and lipid accumulation. We further suggest that the administration of vinpocetine could reduce the incidence of diseases, such as obesity and diabetes, and further clinical studies are warranted to investigate its practical application.

## Supplementary information

Supplementary figure 1

Supplementary figure 2

Supplementary figure 3

Supplementary figure 4

Supplementary figure 5

Supplementary figure 6

Supplementary figure 7
