# Supplementary figures and images for: A PDE1 inhibitor reduces adipogenesis in mice via regulation of lipolysis and adipogenic cell signaling
Source: Exp Mol Med. 2019 Jan 11;51(1):5. doi: 10.1038/s12276-018-0198-7 (PMC6329698; doi:10.1038/s12276-018-0198-7)

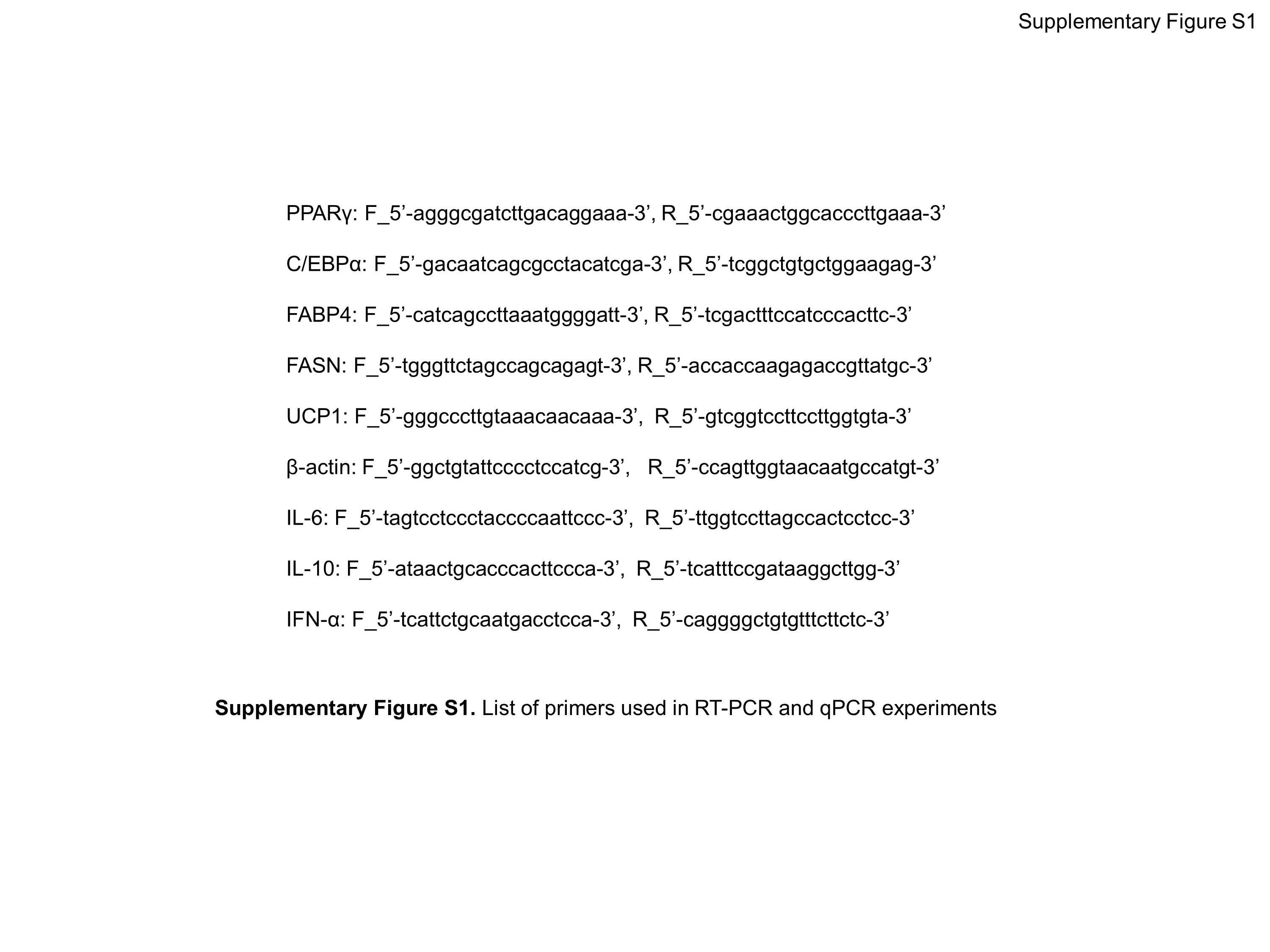

Supplement: Supplementary file 1 — Supplementary figure 1 [file 12276_2018_198_MOESM1_ESM.tif]

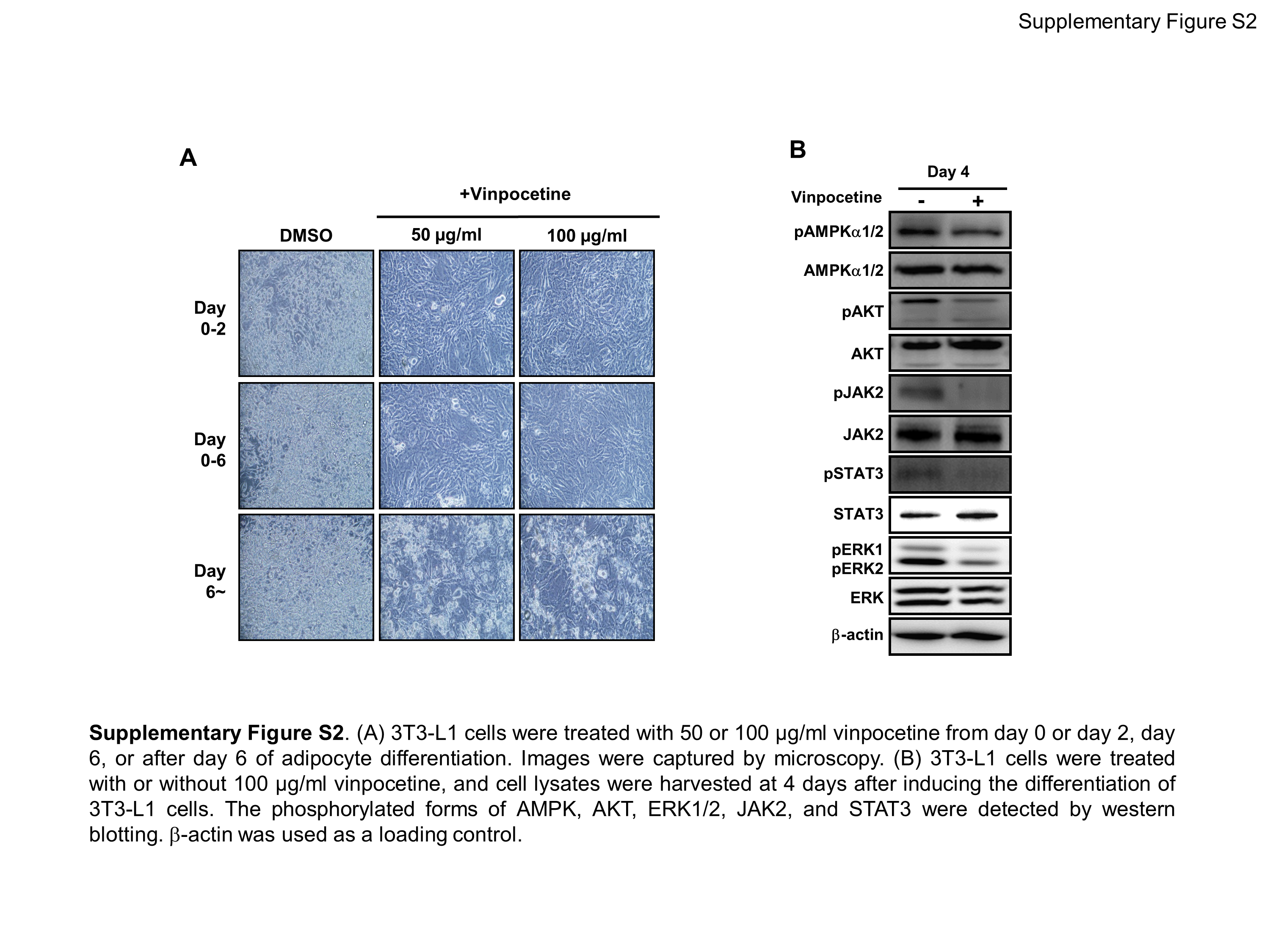

Supplement: Supplementary file 2 — Supplementary figure 2 [file 12276_2018_198_MOESM2_ESM.tif]

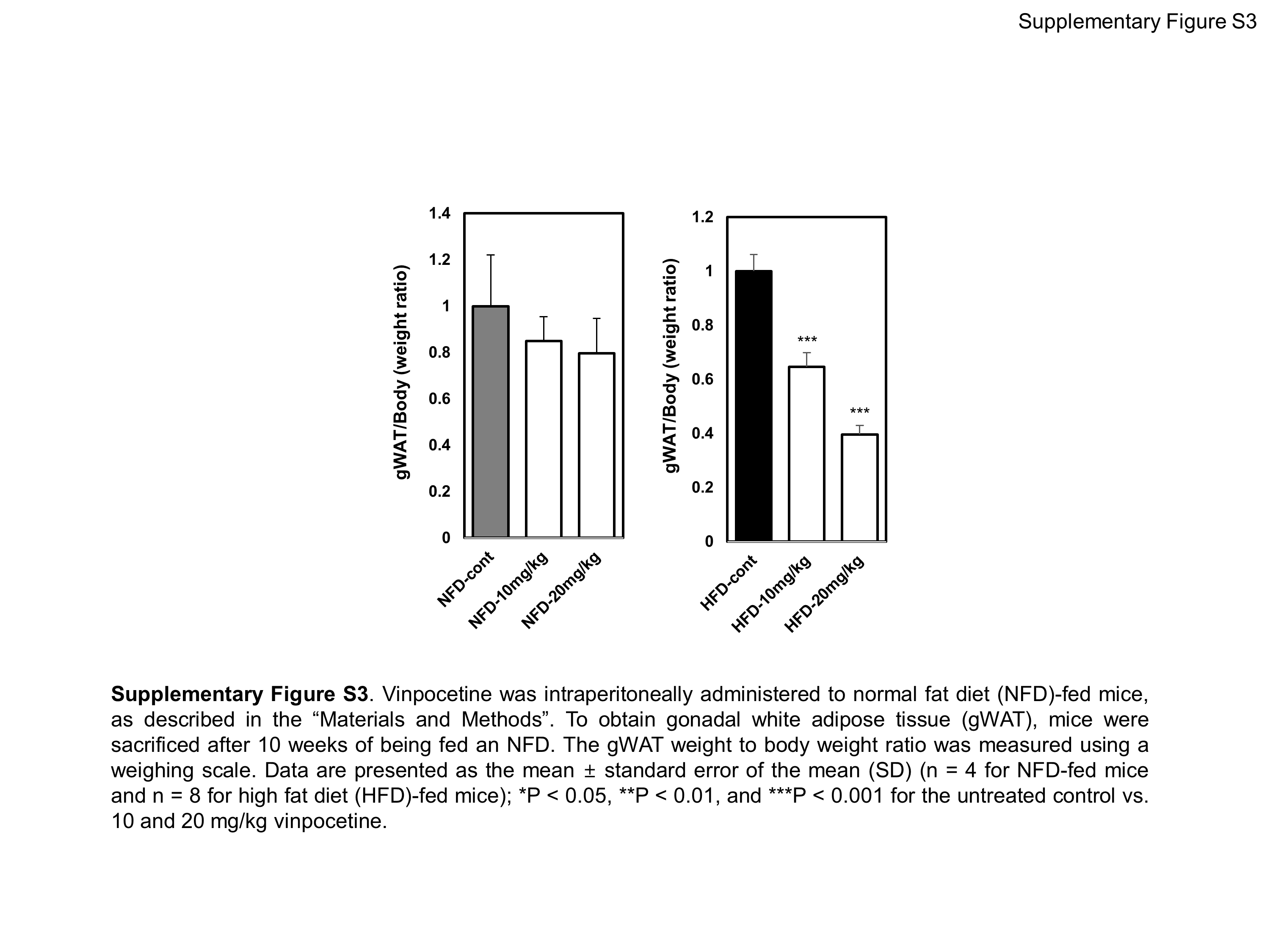

Supplement: Supplementary file 3 — Supplementary figure 3 [file 12276_2018_198_MOESM3_ESM.tif]

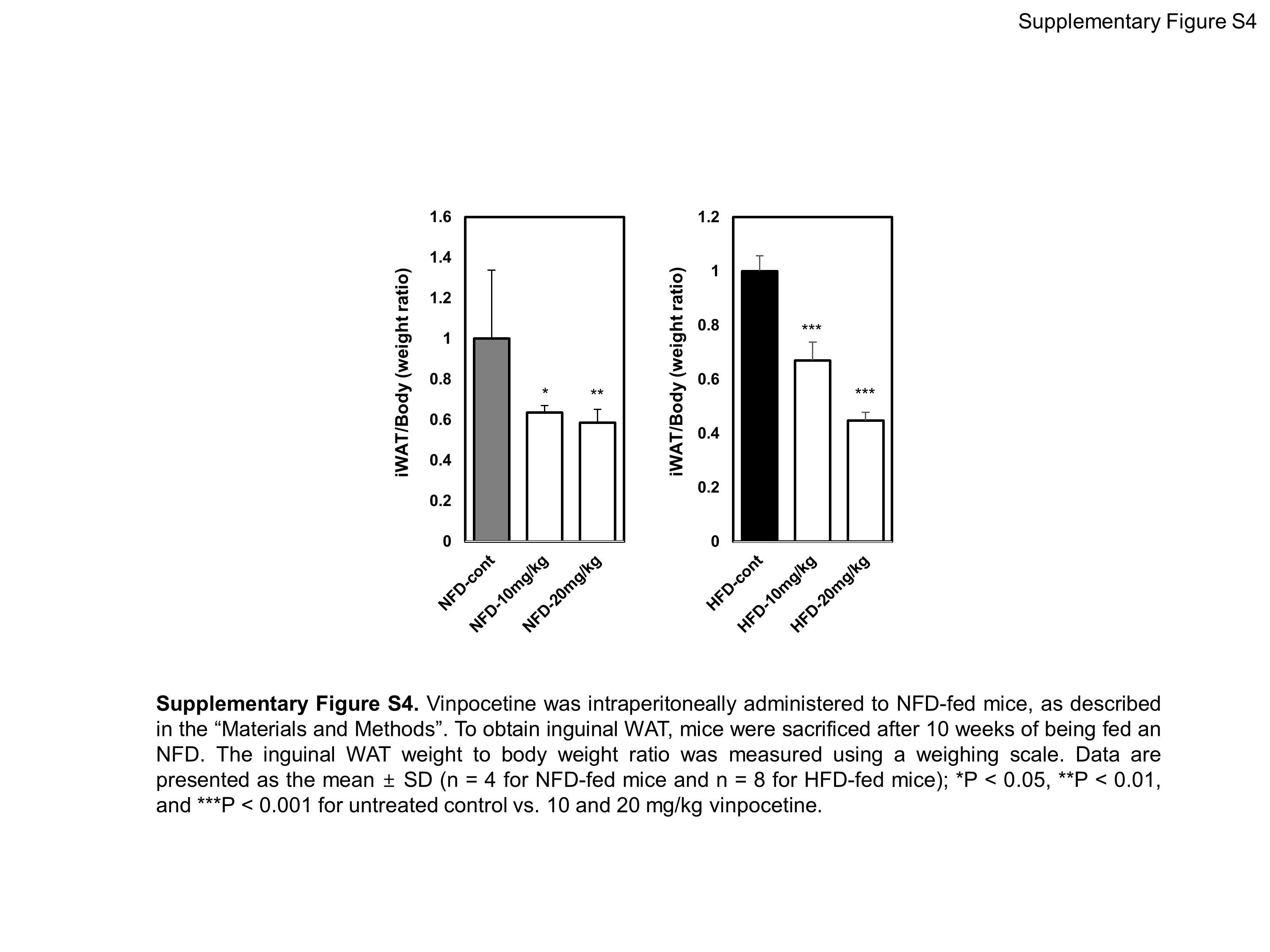

Supplement: Supplementary file 4 — Supplementary figure 4 [file 12276_2018_198_MOESM4_ESM.tif]

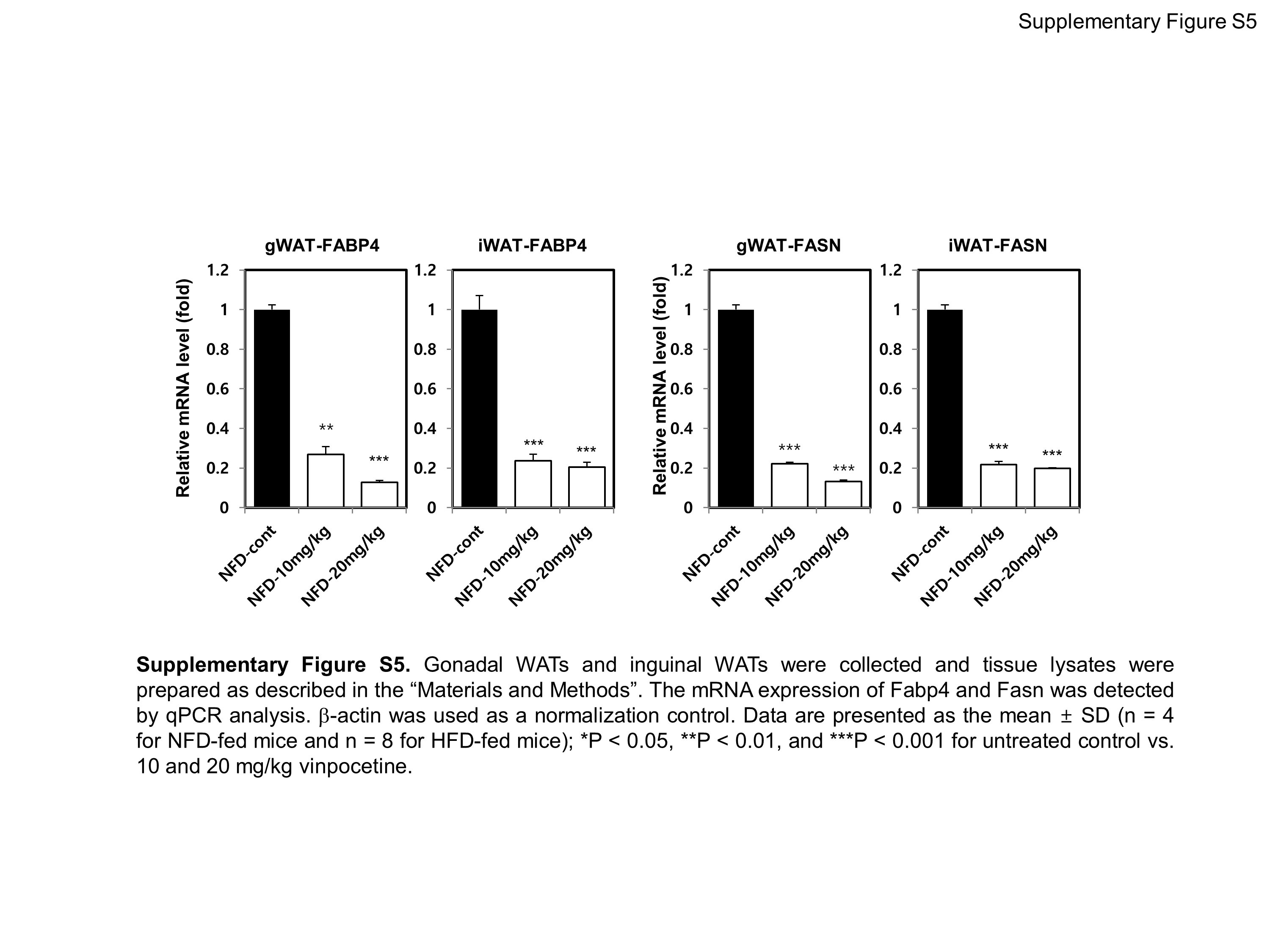

Supplement: Supplementary file 5 — Supplementary figure 5 [file 12276_2018_198_MOESM5_ESM.tif]

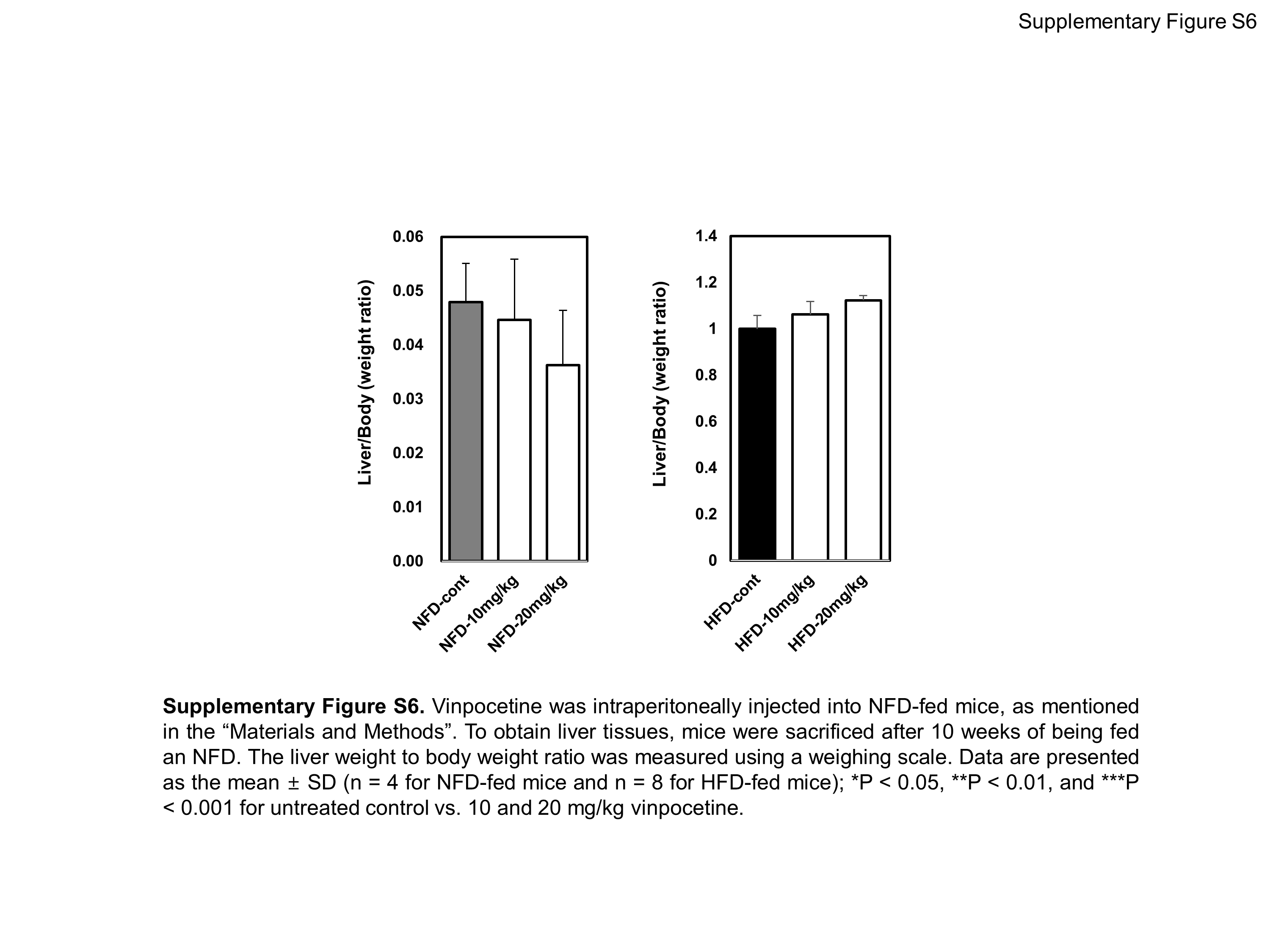

Supplement: Supplementary file 6 — Supplementary figure 6 [file 12276_2018_198_MOESM6_ESM.tif]

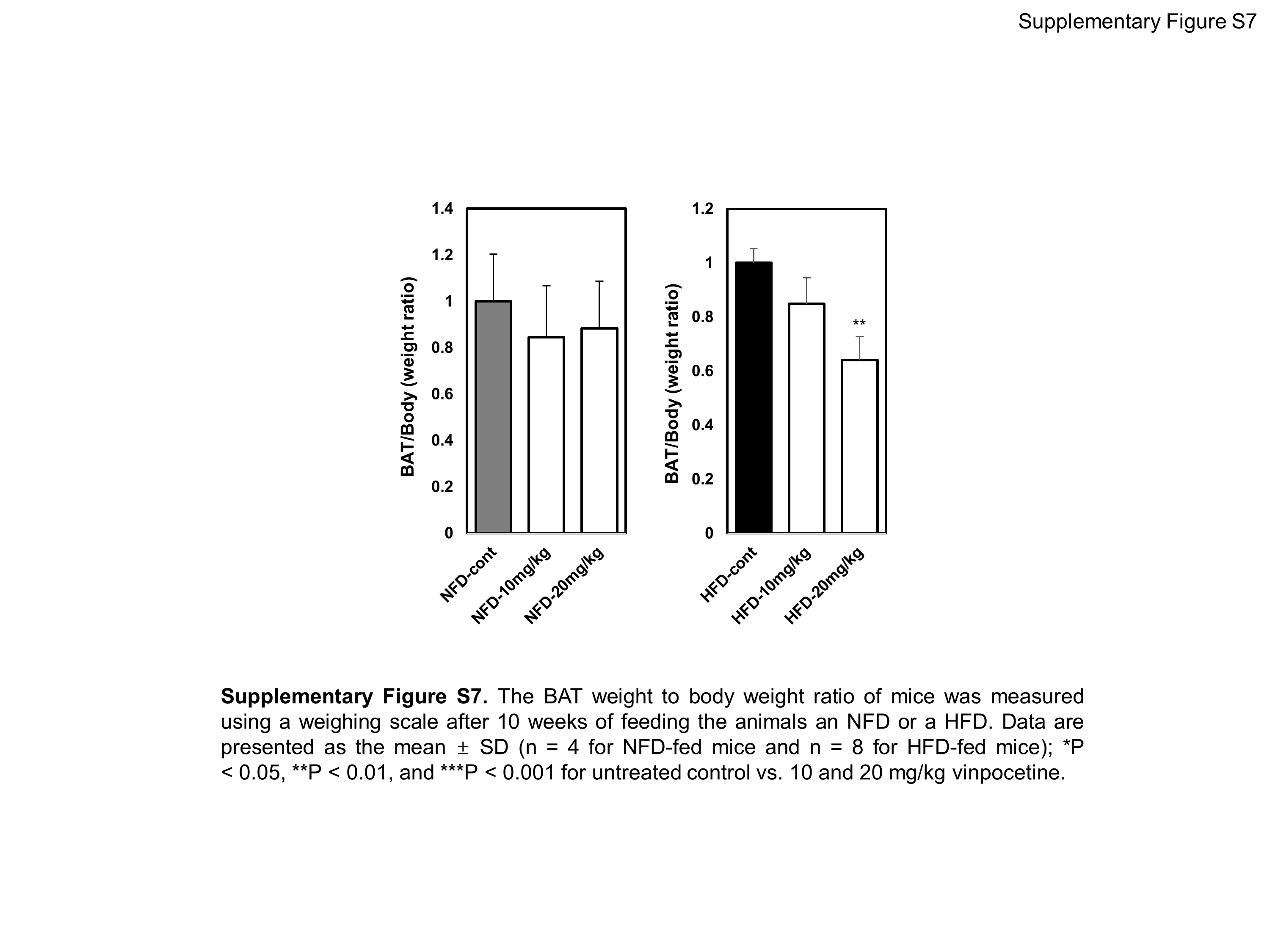

Supplement: Supplementary file 7 — Supplementary figure 7 [file 12276_2018_198_MOESM7_ESM.tif]
